# The IL-1 Receptor Is Required to Maintain Neutrophil Viability and Function During *Aspergillus fumigatus* Airway Infection

**DOI:** 10.3389/fimmu.2021.675294

**Published:** 2021-07-12

**Authors:** Benjamin AWR Ralph, Melanie Lehoux, Hanna Ostapska, Brendan D. Snarr, Alayna K. Caffrey-Carr, Richard Fraser, Maya Saleh, Joshua J. Obar, Salman T. Qureshi, Donald C. Sheppard

**Affiliations:** ^1^ Department of Microbiology and Immunology, McGill University, Montréal, QC, Canada; ^2^ Program in Infectious Diseases and Immunology in Global Health, Centre for Translational Biology, The Research Institute of the McGill University Health Center (RI-MUHC), Montréal, QC, Canada; ^3^ Department of Microbiology & Immunology, Dartmouth College, Lebanon, NH, United States; ^4^ Department of Medicine, McGill University, Montréal, QC, Canada; ^5^ Department of Experimental Medicine, McGill University, Montréal, QC, Canada; ^6^ Department of Biochemistry, McGill University, Montréal, QC, Canada; ^7^ Program in Translation Research in Respiratory Diseases and Department of Critical Care, Centre for Translational Biology, The Research Institute of the McGill University Health Center (RI-MUHC), Montréal, QC, Canada; ^8^ Meakins-Christie Laboratories, McGill University, Montréal, QC, Canada

**Keywords:** *Aspergillus fumigatus*, chronic airway infection, IL-1, G-CSF, neutrophils

## Abstract

*Aspergillus fumigatus* airway infections are associated with increased rates of hospitalizations and declining lung function in patients with chronic lung disease. While the pathogenesis of invasive *A. fumigatus* infections is well studied, little is known about the development and progression of airway infections. Previous studies have demonstrated a critical role for the IL-1 cytokines, IL-1α and IL-1β in enhancing pulmonary neutrophil recruitment during invasive aspergillosis. Here we use a mouse model of *A. fumigatus* airway infection to study the role of these IL-1 cytokines in immunocompetent mice. In the absence of IL-1 receptor signaling, mice exhibited reduced numbers of viable pulmonary neutrophils and increased levels of neutrophil apoptosis during fungal airway infection. Impaired neutrophil viability in these mice was associated with reduced pulmonary and systemic levels of G-CSF, and treatment with G-CSF restored both neutrophil viability and resistance to *A. fumigatus* airway infection. Taken together, these data demonstrate that IL-1 dependent G-CSF production plays a key role for host resistance to *A. fumigatus* airway infection through suppressing neutrophil apoptosis at the site of infection.

## Introduction

Healthy humans inhale hundreds of conidia of the ubiquitous mold *Aspergillus fumigatus* on a daily basis without developing pulmonary disease (1, 2). Elimination of this fungal challenge relies on both the airway mucociliary elevator as well as phagocytosis and killing of conidia by alveolar macrophages and epithelial cells (3, 4). In patients with impaired systemic immunity, such as those undergoing cytotoxic chemotherapy or stem cell transplantation, *A. fumigatus* conidia escape the impaired innate immune responses to germinate and invade tissues, producing a necrotizing, invasive pneumonitis known as invasive aspergillosis (5). In contrast, in patients with chronic pulmonary conditions such as cystic fibrosis or bronchiectasis, the inability to efficiently clear conidia from damaged airways can lead to the development of chronic non-invasive *A. fumigatus* airway infection (6, 7). *A. fumigatus* airway infections are common in chronic pulmonary conditions, with up to 80% of patients with cystic fibrosis having a positive respiratory culture for this fungus at some point in their lives (7, 8). The consequences of chronic *A. fumigatus* airway infections are variable and include asymptomatic colonization, Aspergillus bronchitis (characterized by neutrophilic inflammation), and allergic bronchopulmonary aspergillosis (characterized by severe hypersensitivity to fungal antigens, reactive airway disease and bronchiectasis) (9, 10). The acquisition of airway infection with *A. fumigatus* has been linked to increased rates of hospitalization and worsening airway function in patients with chronic lung disease, even in the absence of a significant allergic response (9, 11). Although numerous studies have probed the pathogenesis of invasive aspergillosis, little is known about the host and fungal factors governing the development and progression of chronic *A. fumigatus* airway infections.

Experimental studies have demonstrated a critical role for the IL-1 cytokine family members IL-1α and -1β, in host resistance to invasive aspergillosis (12, 13). IL-1α is a proinflammatory cytokine and alarmin that is released in response to tissue injury. IL-1α constitutively expressed in pulmonary epithelial cells and can be induced in leukocytes (14, 15). Conversely, IL-1β is not constitutively expressed and is produced and secreted by leukocytes in response to a 2-step mechanism initiated by pathogen recognition or cytokine signaling (16–18). The first step results in the production of inactive pro-cytokine, followed by proteolytic cleavage and activation of the cytokine prior to secretion (17, 19). Both IL-1 cytokines mediate their activity by binding to, and signaling through the IL-1R1 receptor (17, 19, 20). In a high-inoculum challenge immunocompetent mouse model of invasive aspergillosis, IL-1α was reported to promote host resistance to *A. fumigatus* by enhancing the production of chemokine (C-X-C motif) ligand 1 (CXCL1) leading to pulmonary neutrophil recruitment (12). In a corticosteroid-treated immunosuppressed mouse model, impaired IL-1β expression in caspase 1^-/-^ mice or IL-1β^-/-^ mice was associated with increased mortality and reduced numbers of pulmonary neutrophil (13). Recently, we reported increased levels of pulmonary IL-1α and IL-1β in a mouse model of non-invasive *A. fumigatus* airway infection (21), and therefore hypothesized that IL-1 cytokines may also play an important role in mediating protection against non-invasive airway *Aspergillus* infection.

In the current study, we demonstrate that IL-1α and IL-1β secretion is induced in response to experimental *A. fumigatus* airway infection. IL-1R1-deficient (IL-1R1^-/-^) mice exhibited impaired control of fungal growth within the airways in association with lower numbers of pulmonary neutrophils, reduced pulmonary neutrophil viability and higher levels of neutrophil apoptosis. Reduced pulmonary neutrophil viability in IL-1R1^-/-^ mice was associated with reduced production of granulocyte colony stimulating factor (G-CSF). Administration of recombinant G-CSF was sufficient to reduce neutrophil apoptosis, restore the number of viable pulmonary neutrophils, and the augment the resistance of IL-1R1^-/-^ mice to *A. fumigatus* infection to that of wild-type mice. Collectively, these experiments establish the importance of IL-1R1 signaling in the host response against *A. fumigatus* airway infection, and also demonstrate an important role for this pathway in maintaining neutrophil viability through the induction of G-CSF secretion.

## Material and Methods

### Mice

All the animal experiments carried out in this study were approved by the Animal Care Committees of the McGill University Health Centre (Animal Usage Protocol 7609) and the Dartmouth College Institutional Animal Care and Use Committee (Protocol #obar.jj.1). Age and sex matched C57BL/6 mice were purchased from Charles River Laboratories. IL-1R1^-/-^, mice were provided by Dr. Maya Saleh (McGill University, Montreal, Canada).

### Agar Bead Infection

Agar beads containing *A. fumigatus* conidia were prepared as described previously (21). Prior to infection, the inoculum was verified by homogenization and quantitative culture of an aliquot of each bead preparation. Eight to 10-week-old mice were anesthetised with isoflurane and then endotracheally infected with 50 µL of an agar bead suspension containing 2.5 x 10^6^ conidia. Mice treated with IL-1 receptor antagonist (IL-1Ra) were intraperitoneally administered 200µg of Kineret^®^ (Sobi, Inc) daily, commencing on day -1 relative to infection. Mice treated with recombinant human G-CSF (rhG-CSF) were intraperitoneally administered 250μg/kg of rhG-CSF (Amgen Inc.) daily, commencing on the day of infection. At the indicated time points, mice were euthanized with isoflurane and CO_2_, their lungs harvested and then digested with 150U/mL of collagenase for 60 minutes at 37°C (for pulmonary leukocyte population analysis), homogenized using a polytron tissue homogenizer (for fungal burden determination) as previously described (21), or finely diced using two scalpels (for *ex vivo* cytokine elaboration assays).

### Fungal Burden

Pulmonary fungal burden by quantified by measuring the galactomannan content of lung digests or homogenates as we have done previously (21). Briefly, lung homogenates were diluted in double distilled water (ddH_2_0) and galactomannan was quantified according to the Platelia™ Aspergillus enzyme immunoassay kit (Bio-Rad) manufacturer’s instructions.

### *Ex Vivo* Cytokine Production

Three days after infection, mice were euthanized and their lungs harvested. Using two scalpels, lungs were finely diced and suspended in 5 mL of RPMI (Wisent) containing 10% FBS (Wisent) and 1% penicillin/streptomycin (Wisent). Diced lungs were incubated on a rotating platform (Wisent) at 37°C and 5% CO_2_ for 24 hours, after which the supernatants were collected and cytokine secretion assessed by commercial ELISA (Thermo Fisher Scientific Inc.) for CXCL1, G-CSF, IL-1α, and IL-1β.

### Pulmonary Cell Population Analysis

Following collagenase digestion, lungs cells were passed through a cell strainer and red blood cells were lysed with ammonium-chloride-potassium buffer as previous described (21). The resulting cells were stained for viability using Fixable Viability Dye eFluor506 (eBioscience), and for CD45, CD11b, CD11c, Ly6G, CD3, CD19, and SiglecF expression (BD Bioscience) (21). Data was acquired on an LSR Fortessa (BD Bioscience) and analyzed using FlowJo v10.0.7r2 (FlowJo, LLC). Neutrophils were defined as CD45^+^CD11b^+^CD11c^-^Ly6G^+^, alveolar macrophages as CD45^+^CD11c^+^CD11b^low^SiglecF^+^, eosinophils as CD45^+^CD11c^-^SiglecF^+^CD11b^+^, and lymphocytes were the sum of T-cells (CD45^+^CD11c^-^ly6G^-^CD3^+^) and B-cells (CD45^+^CD11c^-^ly6G^-^CD19^+^) (**Supplementary Figure 1**). The absolute number of cells per lung was calculated by the addition of counting beads to samples (Thermo Fisher Scientific Inc.).

### Lung Histopathology

Harvested lungs were inflated with 10% buffered formalin and submerged in formalin for up to 48 hours before being embedded in paraffin blocks and sectioned. Sections were stained with periodic acid–Schiff (PAS) or hematoxylin and eosin (H&E) to visualize fungi or host cells, respectively. For cleaved caspase 3 detection by immunohistochemisty, antigen retrieval, staining, and signal developing was performed using an automated immunostainer (Bond RX, Leica Biosystems). Anti-cleaved caspase 3 antibody (Abcam) was diluted and incubated for 30 minutes at room temperature. Visualization was performed using the Bond Polymer Refine Detection kit (Leica Biosystems) following the manufacturer’s instructions.

### Density of Hyphae in Beads

Using ImageScope (Leica Biosystems) software, representative beads of equal size were selected from scanned sections of PAS-stained lungs. Beads were outlined and analyzed using the Positive Pixel Count V9 algorithm in the software. The average pixel intensity for each bead was normalized to the average pixel intensity of the beads from the C57BL/6 infected mice.

### Neutrophil Antifungal Activity

Mouse bone marrow neutrophils were isolated by negative selection using a magnetic bead isolation kit (Miltenyl Biotec). Following isolation, 2 x 10^5^ neutrophils were suspended in DMEM (Wisent) containing 10% FBS (Wisent) and 1% Penicillin/Streptomycin (Wisent) (cDMEM) and added to microtiter wells containing 3 x10^3^ *A. fumigatus* germlings that had been pre-grown for 8 hours. After 16 hours of co-incubation, wells were washed with sterile water to lyse the remaining neutrophils, and the remaining hyphae stained for 1 minute with 50µL of a filter sterilized 1mg/mL calcofluor (Sigma) white in 5% KOH (Fisher Scientific) solution. Hyphae were washed with sterile water 3 times then staining was quantified using a fluorometer with 360nm excitation and 440nm emission.

### Neutrophil Viability Assays

For quantification of neutrophil death by measurement of lactate dehydrogenase (LDH) release, 24-well plates were inoculated with 2 X 10^6^ bone-marrow isolated neutrophils and incubated for 24 hours. 50µL of sham agar beads or beads containing total of 2.5 x 10^6^ conidia were plated in 2mL of RPMI (Wisent) containing 10% FBS (Wisent) and 1% penicillin/streptomycin (Wisent) (cRPMI). The degree of neutrophil death was determined by quantification of LDH activity (Promega) in culture supernatants was determined, following the manufacturer’s instructions.

For quantification of neutrophil viability and apoptosis by flow cytometry, 2 X 10^6^ neutrophils isolated from mouse bone marrow were incubated in 2mL of cRPMI in a 24-well plate for 24 hours with or without 600ng/mL recombinant human G-CSF (rhG-CSF, Amgen Inc.) as indicated. Neutrophils were then stained for apoptosis and viability using phycoerythrin labelled Annexin V (BD Bioscience) and the dye 7-AAD in binding buffer (0.01 M Hepes (pH 7.4), 0.14 M NaCl, 2.5 mM CaCl2). The degree of staining was quantified by flow cytometry (22).

### Quantification of Anti-Apoptotic Gene Expression

In a 24-well plate, 2 X 10^6^ neutrophils were incubated in 2mL of cRPMI for 24 hours with or without the addition of 600ng/mL rhG-CSF (Amgen Inc.). Neutrophils were collected and RNA extracted by trizol as previously described (23). Anti-apoptotic gene mRNA levels were quantified as previously described using TaqMan probes (Thermo Fisher Scientific Inc.) for BCL-xL (Mm00437783_m1) and MCL-1 (Mm01257351_g1), and endogenous reference genes 18s RNA (Mm03928990_g1) and GAPDH (Mm99999915_g1) (24).

### Reactive Oxygen Species (ROS) Detection

In 1.5ml tubes, 2 X 10^6^ neutrophils/mL were treated with the ROS detection dye CM-H2DCFDA (Thermo Fisher Scientific Inc.) at a concentration of 2µM for 45 minutes at 37°C. Cells were washed and resuspended at a concentration of 2 x 10^5^ cells/well in 200µL of cRPMI with, 2µM phorbol myristate acetate (PMA). Relative fluorescence was measured at 45 minutes with an excitation of 492nm and emission of 527nm.

### Statistics Analysis

GraphPad Prism software was used for all the statistical analysis. Significance was determined by Student’s t-test, one-way ANOVA using a Bonferroni post-test, or two-way ANOVA using Tukey’s post-test as indicated in the relevant Figure legend.

## Results

### IL-1α and IL-1β Secretion Is Induced by *A. fumigatus* Airway Infection

**Figure 1:**
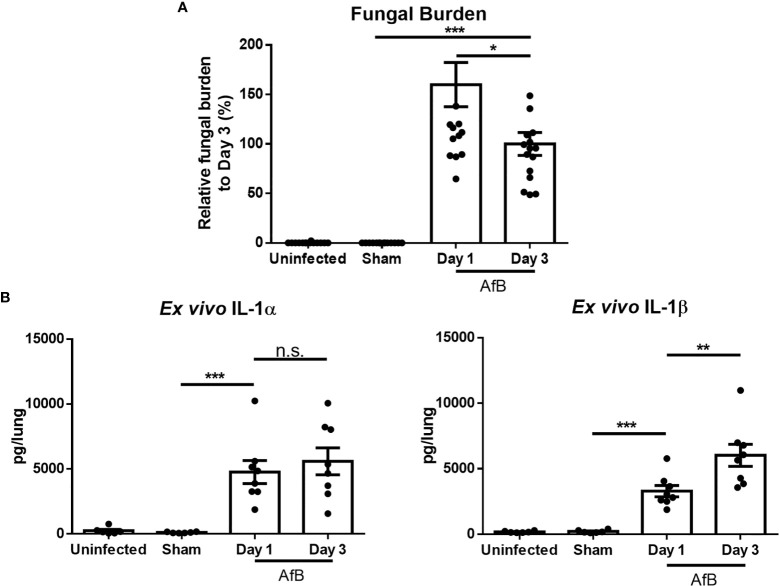
*A. fumigatus* airway infection induces the production of IL-1α and IL-1β. C57BL/6 mice were infected intratracheally with sterile agar beads (Sham) or agar beads containing *A. fumigatus* conidia (AfB). **(A)** Pulmonary fungal burden as measured by galactomannan Platelia™ ELISA at the indicated time points after infection with *A. fumigatus*-containing beads. **(B)** Pulmonary *ex vivo* IL-1 cytokine secretion as determined by ELISA one and three days after infection. Results are presented as the means ± SEM of groups of at least 7 mice across 2 experiments, *, ** and *** indicates significantly different as compared with Sham mice, p ≤ 0.05, p ≤ 0.01 and p ≤ 0.001 respectively (one-way ANOVA t-test). n.s., not significant.

Previously, we reported that levels of the proinflammatory cytokines IL-1α and IL-1β are elevated in lung homogenates of immunocompetent mice during non-invasive *A. fumigatus* airway infection (21). However, as IL-1α is constitutively expressed intracellularly and IL-1β is first produced intracellularly prior to secretion, the measurement of these cytokines in lung homogenates is unable to distinguish secreted, biologically active, IL-1α and IL-1β from intracellular reservoirs of these molecules. Bronchoalveolar lavage sampling of infected airways is of limited value in this mouse model, as histopathologic examination of lungs reveals the presence of intrabronchial lesions that frequently obstruct the airways, reducing the ability to sample the distal airways. Therefore, to specifically quantify secreted IL-1α and IL-1β, bead-infected lungs were cultured *ex vivo* and secreted cytokines measured by ELISA. *Ex vivo* culture of lungs after 1 and 3 days of airway infection revealed the secretion of high levels of IL-1α and IL-1β in lungs of mice with *A. fumigatus* airway infection, as compared to lungs from uninfected mice or mice infected with sterile agar (sham) beads (**Figure 1**). The levels of secreted IL-1 cytokines increased from day 1 to day 3 of infection, while the fungal burden decreased between these time points. In light of the higher levels of IL-1 cytokines at day 3 of infection, this time point was chosen for further studies.

### IL-1R Signaling Mediates Resistance to *A. fumigatus* Airway Infection

**Figure 2:**
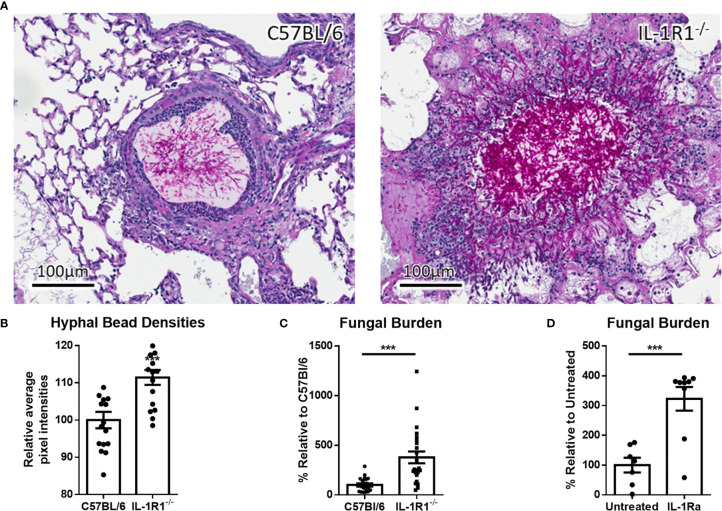
Loss of IL-1 receptor signaling results in increased susceptibility to *A. fumigatus* airway infection. **(A)** PAS stained tissue sections of lungs from wild-type or IL-1R1^-/-^ mice 3 days after infection with *A. fumigatus*-containing beads. **(B)** Relative hyphal bead density as measured by average intra-bead pixel intensity in ImageScope. Results represent means ± SEM of 16 beads from 3 different mice, ***p ≤ 0.001 (Student’s t-test). **(C)** Pulmonary fungal burden of C57BL/6 and IL-1R1^-/-^ or **(D)** C57BL/6 mice treated with IL-1Ra 3 days after infection with *A. fumigatus* beads. Fungal burden was measured by galactomannan EIA. Results represent the means ± SEM of groups of at least 9 mice across 2 experiments, ***p ≤ 0.001 (Student’s t-test).

IL-1α and IL-1β signal through binding to the IL-1R1 receptor (19). Therefore, to determine the role of IL-1 cytokines in the pathogenesis of *A. fumigatus* airway infection, the susceptibility of IL-1R1-deficient and wild-type C57BL/6 mice to airway infection with *A. fumigatus* was compared. After 3 days of infection, histopathologic examination of lung tissues from infected animals revealed that, unlike wild-type mice, IL-1R1^-/-^ mice were unable to contain hyphae within the agar beads (**Figure 2A**). In addition, pixel quantification of fungal lesions demonstrated an increase density of fungal hyphae within pulmonary lesions of IL-1R1^-/-^ mice as compared to wild-type animals (**Figure 2B**). Consistent with these observations, IL-1R1^-/-^ mice displayed a significantly higher fungal burden than did wild-type mice, as measured by pulmonary galactomannan content (**Figure 2C**).

To confirm these findings, the effects of IL-1R1 blockade with IL-1 receptor antagonist (IL-Ra) on pulmonary fungal burden during *A. fumigatus* airway infection were assessed. Treatment of wild-type mice with IL-1Ra resulted in an increase in pulmonary fungal burden similar to that which was observed in IL-1R1^-/-^ mice (**Figure 2D**). These findings suggest that IL-1 receptor signaling plays a role in mediating resistance to *A. fumigatus* airway infection.

### IL-1R1-Deficient Mice Have Reduced Number of Pulmonary Neutrophils and Alveolar Macrophages During Non-Invasive *A. fumigatus* Airway Infection

**Figure 3:**
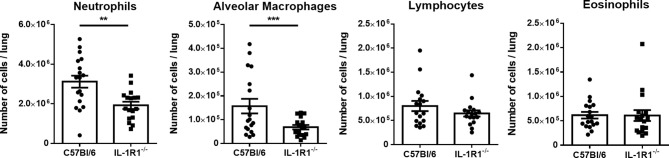
Neutrophils and macrophages are less abundant in the lungs of IL-1 receptor-deficient mice during *A. fumigatus* airway infection. Total pulmonary neutrophils, alveolar macrophages, lymphocytes, and eosinophils were determined by flow cytometry analysis of C57BL/6 and IL-1R1^-/-^ collagenase digested lungs of the indicated mouse strains 3 days after infection with *A. fumigatus* beads. Results represent means ± SEM of groups of n > 15 mice across 2 experiments, **p < 0.01, ***p ≤ 0.001 (Student’s t-test).

**Figure 4:**
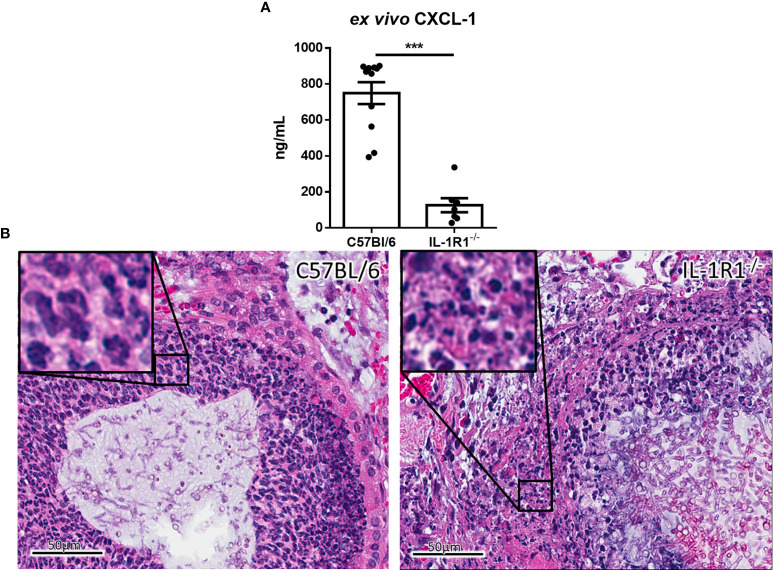
IL-1 receptor-deficient mice exhibit reduced chemokine production and increased leukocyte cell death during *A. fumigatus* airway infection. **(A)** Pulmonary *ex vivo* CXCL1 cytokine secretion as determined by ELISA three days after infection. Results represent means ± SEM of groups of n ≥ 7 mice across 2 experiments, ***p ≤ 0.001 (Student’s t-test). **(B)** H&E stained tissue sections of lungs from C57BL/6 or IL-1R1^-/-^ mice 3 days after infection with *A. fumigatus*-containing beads.

To determine whether the increased fungal burden in IL-1R1^-/-^ mice may result from impaired IL-1R-dependent pulmonary recruitment of neutrophils or other leukocytes, the leukocyte populations within the lungs of IL-1R1^-/-^ and wild-type mice were quantified after 3 days of airway infection. When compared to wild-type mice, IL-1R1^-/-^ mice were found to have significantly lower numbers of pulmonary neutrophils and alveolar macrophages but not eosinophils, B or T lymphocytes (**Figure 3**). Consistent with the reduced abundance of pulmonary neutrophils and with previous reports (12, 25), infected IL-1R1^-/-^ mice were found to have significantly lower pulmonary levels of the neutrophil attracting chemokine CXCL1 as compared to wild-type mice (**Figure 4A**). This difference in neutrophil abundance was not observed when comparing the bone marrow of wild-type and IL-1R1^-/-^ mice, suggesting that lack of IL-1 receptor signaling does result in a baseline impairment in neutrophil production (**Supplementary Figure 2**).

On histopathological examination of pulmonary tissues of IL-1R1^-/-^ mice, fewer intact neutrophils were observed surrounding fungal lesions as compared with wild-type animals (**Figure 4B**). In addition, marked changes in neutrophil morphology were observed in the lungs of IL-1R1^-/-^ mice with increased nuclear fragmentation and karyorrhexis, suggesting that the reduced number of neutrophils found in the lungs of IL-1R1^-/-^ mice may also reflect accelerated neutrophil death rather than simply a defect in neutrophil recruitment.

### IL-1R1^-/-^ Neutrophils Exhibit Increased Apoptosis *In Vivo* and *In Vitro*

**Figure 5:**
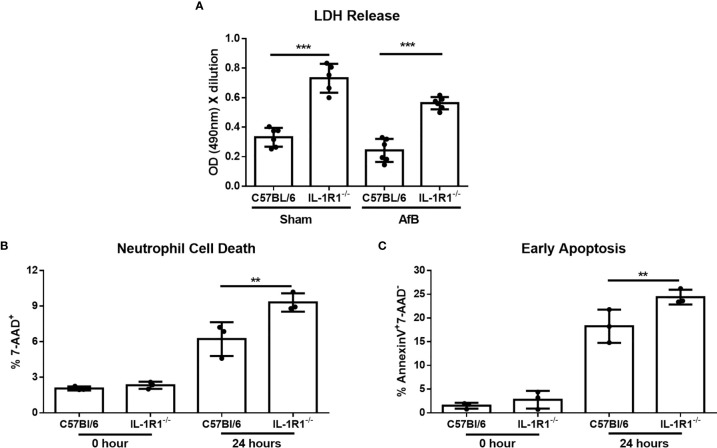
IL-1 receptor signaling reduces neutrophil apoptosis *in vitro*. **(A)** Neutrophil cell death as measured by LDH release following co-culture of bone-marrow isolated neutrophils from the indicated mouse strains with sterile agar beads (Sham) or *A. fumigatus* conidia-containing beads (AfB) for 24 hours. Results represent mean ± SD of 2 independent experiments, ***p ≤ 0.001, (one-way ANOVA test). **(B)** Neutrophil cell death as measured by 7-AAD staining and flow cytometry analysis of bone-marrow isolated neutrophils from the indicated mouse strains directly after isolation (0 Hour) or after 24 hours of culture in 10% FBS supplemented RPMI. Results represent means ± SD of 3 independent experiments, **p ≤ 0.01, (one-way ANOVA test). **(C)** Apoptosis of bone-marrow isolated neutrophils from the indicated mouse strains as measured by Annexin V staining either directly after isolation (0 hour) or after 24 hours of culture. Results represent mean ± SD of 3 independent experiments, **p ≤ 0.01, (one-way ANOVA test).

To determine if IL-1R1^-/-^ neutrophils exhibit reduced viability in the presence of *A. fumigatus*, neutrophils isolated from the bone marrow of IL-1R1^-/-^ and wild-type mice were co-incubated with *A. fumigatus-*containing agar beads for 24 hours. Lactate dehydrogenase (LDH) release was measured in culture supernatants as a surrogate measurement for cell death. IL-1R1^-/-^ neutrophils co-cultured with *A. fumigatus*-containing beads released significantly higher amounts of LDH than did wild-type neutrophils, suggesting that IL-1R1^-/-^ neutrophils exhibited increased levels of cell death. Interestingly, a similar increase in LDH release by IL-1R1^-/-^ neutrophils was observed in the presence of sterile agar beads, suggesting that this viability defect is not *Aspergillus*-specific (**Figure 5A**).

**Figure 6:**
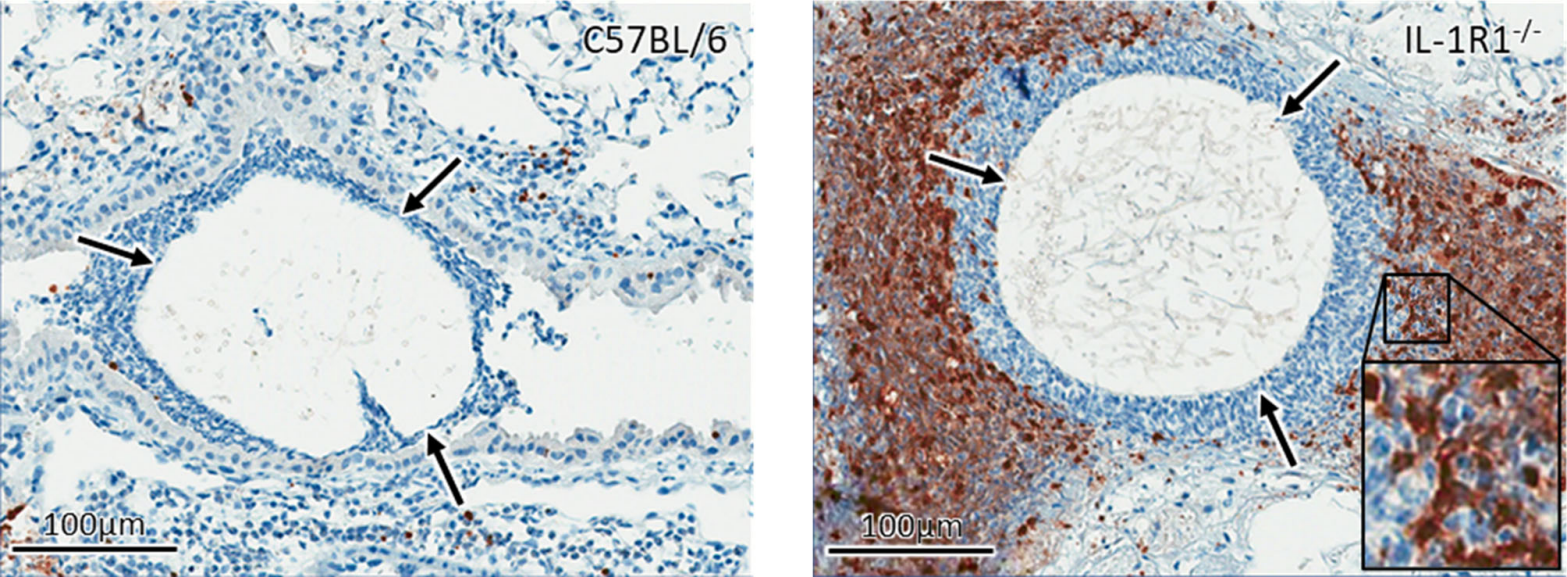
IL-1 receptor signaling reduces apoptosis *in vivo*. Active caspase 3 immunohistochemistry staining (brown) of lungs from wild-type and IL-1R1^-/-^ mice 3 days after infection with *A. fumigatus*-containing beads (arrow).

To confirm these findings and determine if the increased cell death of IL-1R1^-/-^ neutrophils is due to accelerated apoptosis, neutrophils isolated from the bone marrow of wild-type and IL-1R1^-/-^ mice were incubated for 24 hours, and their viability and level of apoptosis assessed by 7-AAD and Annexin V staining, respectively. Consistent with the results of the LDH release assay, IL-1R1^-/-^ neutrophils displayed increased levels of cell death, as measured by 7-AAD^+^ staining (**Figure 5B**). IL-1R1^-/-^ neutrophils also exhibited higher levels of early apoptosis, as measured by 7-AAD^-^ AnnexinV^+^ staining (**Figure 5C**). To determine if IL-1R1^-/-^ neutrophils also exhibit increased apoptosis *in vivo* during an airway infection, immunohistochemistry staining for cleaved caspase 3 was performed on sections of lungs from infected IL-1R1^-/-^ and wild-type mice. Caspase 3 staining was markedly higher in cells surrounding fungal lesions in IL-1R1^-/-^ mice as compared with wild-type mice (**Figure 6**). Consistent with the results of flow cytometry analysis of infected lungs, the majority of these cells exhibited multi-lobed nuclei characteristic of neutrophils. Taken together these data suggest that IL-1R1^-/-^ neutrophils exhibit reduced viability and increased apoptosis during fungal infection, which may contribute to reduced ability to control airway infection.

### IL-1R1 Signaling Is Required for Neutrophil Antifungal Activity and IL-17 Production

**Figure 7:**
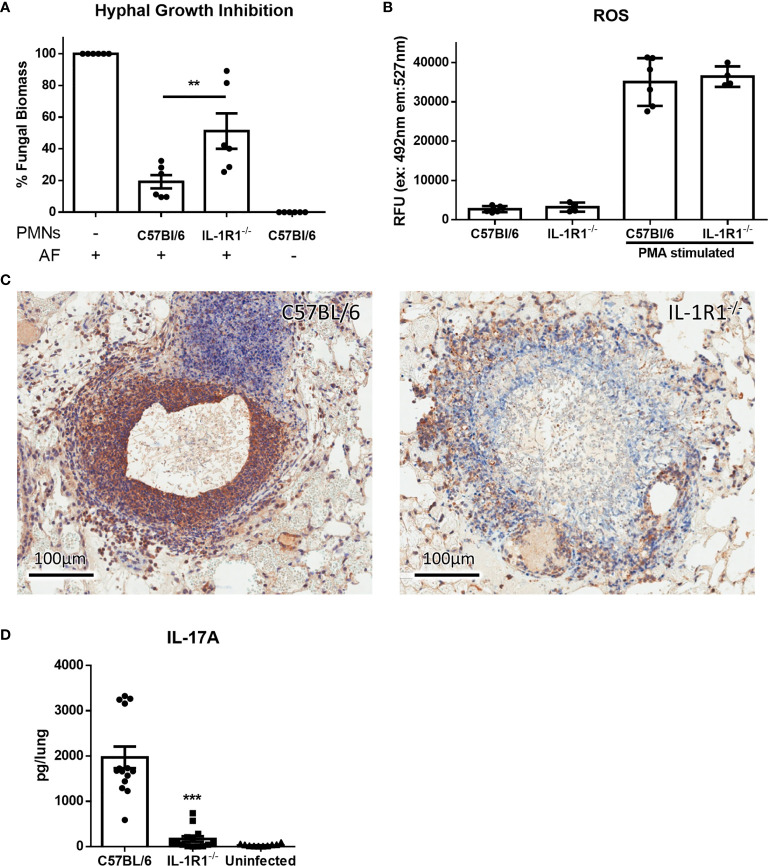
IL-1 receptor-deficient neutrophils have impaired antifungal activity. **(A)** Fungal biomass was measured by calcofluor white staining following 16 hours of co-culture with neutrophils isolated from the bone marrow of the indicated mouse strains. Results represent the mean ± SD of 6 independent experiments, **p ≤ 0.01, (one-way ANOVA test). **(B)** ROS production of bone marrow isolated neutrophils of the indicated mouse strains stimulated with PMA as measured by CM-H2DCFDA based assay kit. Results represent the mean ± SD of 2 independent experiments (one-way ANOVA test). **(C)** IL-17 immunohistochemistry staining (brown) of lungs from wild-type and IL-1R1^-/-^ mice 3 days after infection with *A. fumigatus*-containing beads. **(D)** *Ex vivo* IL-17 production in lungs of the indicated mouse strains infected for 3 days with *A. fumigatus* beads. Results represent the mean ± SEM of groups of at least 10 mice across 2 experiments, ***p ≤ 0.001, (one-way ANOVA test).

In light of their increased rate of apoptosis and cell death, we hypothesized that IL-1R1^-/-^ neutrophils likely exhibit reduced ability to kill *A. fumigatus* hyphae. To test this hypothesis, neutrophils from IL-1R1^-/-^ and wild-type mice were co-cultured with *A. fumigatus* hyphae for 16 hours and the residual fungal biomass quantified using calcofluor white. IL-1R1^-/-^ neutrophils were found to have reduced capacity to restrict *A. fumigatus* growth as compared to wild-type neutrophils (**Figure 7A**). To determine whether the reduced antifungal capacity of these neutrophils was due to an intrinsic impaired ability of the neutrophils to produce the effector ROS, *in vitro* ROS production by bone marrow isolated neutrophils was quantified. Neutrophils from wild-type and IL-1R1^-/-^ mice exhibited similar levels of ROS production (**Figure 7B**). These data suggest that the decreased viability of IL-1R1^-/-^ neutrophils likely results in the impaired ability to control fungal growth rather than an intrinsic defect in ROS-mediated killing.

In addition to mediating direct fungal killing, production of IL-17 by neutrophils has been reported to contribute to the control of mucosal *Aspergillus* infection (26). Immunohistochemistry staining of lung tissue sections confirmed that IL-17 staining, which was localized to the proximal inflammatory cells surrounding fungal lesions in lungs of WT mice, was markedly reduced in the lungs of IL-1R1^-/-^ mice (**Figure 7C**).Therefore, to determine if IL-1R1^-/-^ neutrophils are also defective in the production of this cytokine, the *ex vivo* release of IL-17 from lungs of WT and IL-1R1^-/-^ mice infected with *A. fumigatus* containing agar beads was measured. IL-17 secretion from lungs of IL-1R1^-/-^ mice was markedly reduced as compared to WT mice (**Figure 7D**).

### G-CSF Supplementation Restores IL-1R1^-/-^ Neutrophil Viability and Results in Fungal Burden in IL-1R1^-/-^ Mice Comparable to That of Wild-Type

**Figure 8:**
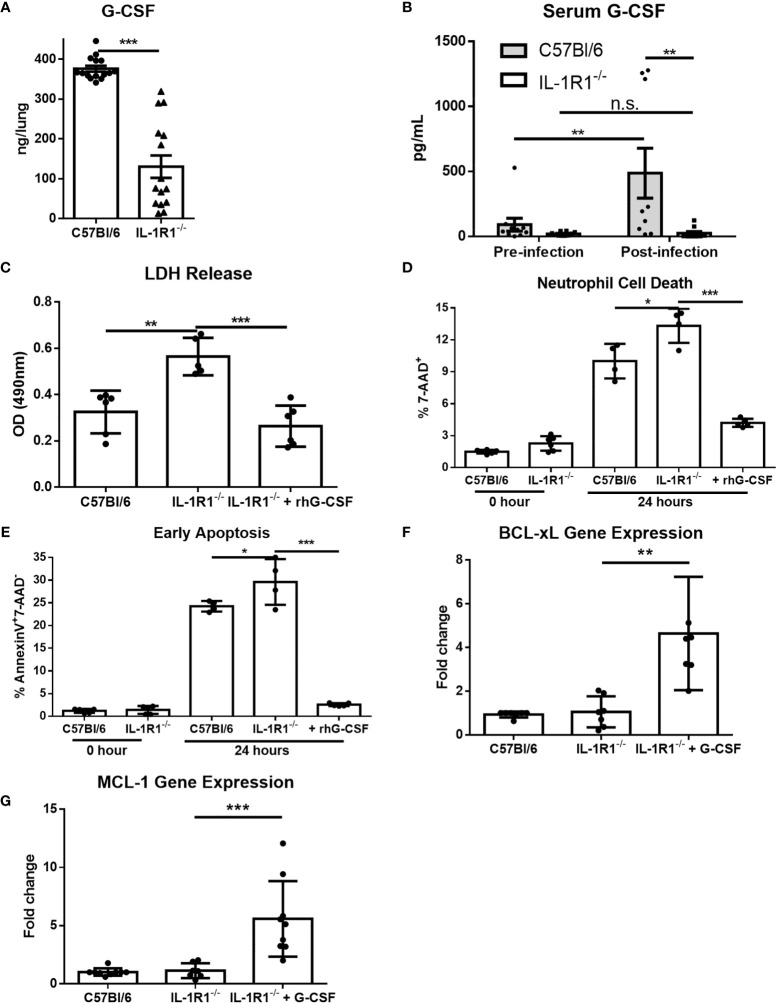
G-CSF secretion is IL-1 receptor-dependent, and reduces neutrophil cell death and increases anti-apoptotic gene expression. **(A)** Pulmonary G-CSF production in lungs of the indicated mouse strains infected for 3 days with *A. fumigatus* beads. Results represent the mean ± SEM of groups of at least 16 mice across 2 experiments, ***p ≤ 0.001, (Student’s t-test). **(B)** G-CSF quantified from the serum of the indicated strains of mice before and 3 days after infection with *A. fumigatus*. Results represent the mean ± SEM of groups of at least 10 mice, **p ≤ 0.01, (two-way ANOVA). **(C)** Neutrophil cell death from bone marrow isolated neutrophils of the indicated strains as measured by LDH release with or without 24 hours incubation with 600ng/mL of G-CSF as indicated. Results represent mean ± SD of 2 independent experiments, **p ≤ 0.001 and ***p ≤ 0.001, (one-way ANOVA test). **(D)** Neutrophil cell death as measured by 7-AAD staining and flow cytometry analysis directly after isolation (0 Hour) or after 24 hours of culture in 10% FBS supplemented RPMI. Cultures were supplemented with 600ng/mL of G-CSF where indicated. Results represent means ± SD of 2 independent experiments, *p ≤ 0.05, (one-way ANOVA test). **(E)** Neutrophil apoptosis as measured by Annexin V staining either directly after isolation (0 hour) or after 24 hours of culture with or without supplementation with 600ng/mL of G-CSF. Results represent mean ± SD of 2 independent experiments, **p ≤ 0.01, (one-way ANOVA test). **(F, G)** Fold change in gene expression of anti-apoptotic genes BCL-xL and MCL-1 as measured by qPCR from bone marrow isolated neutrophils after 24 hours of culture. IL-1R1^-/-^ neutrophils were also treated with 600ng/mL of G-CSF. Results represent mean ± SD of 3 independent experiments, **p ≤ 0.01, (one-way ANOVA test).

G-CSF is a cytokine produced in response to IL-1 receptor signaling which acts specifically on neutrophils to promote differentiation, activation, and viability (27–29). We therefore hypothesized that the decreased viability of IL-1R1^-/-^ neutrophils in *A. fumigatus* airway infection may be due in part to reduced levels of G-CSF. Consistent with this hypothesis, the level of G-CSF secreted *ex vivo* was significantly reduced in infected lungs from IL-1R1^-/-^ mice, as compared to those from wild-type mice (**Figure 8A**). Similarly, IL-1R1^-/-^ mice were found to have significantly lower serum G-CSF levels (**Figure 8B**). To explore the functional significance of this reduced G-CSF production, the effects of G-CSF supplementation on the viability of neutrophils isolated from the bone marrow of IL-1R1^-/-^ and wild-type mice was tested. Treatment with G-CSF inhibited apoptosis and restored the viability of IL-1R1^-/-^ neutrophils to levels comparable to those seen with wild-type neutrophils (**Figures 8C–E**). Consistent with these observations, G-CSF treatment of IL-1R1^-/-^ neutrophils resulted in higher expression levels of the anti-apoptotic genes, BCL-xL and MCL-1, as determined by RT-qPCR (**Figures 8F, G**). These data suggest that *in vitro* supplementation of G-CSF restores neutrophil viability by suppressing apoptosis.

**Figure 9:**
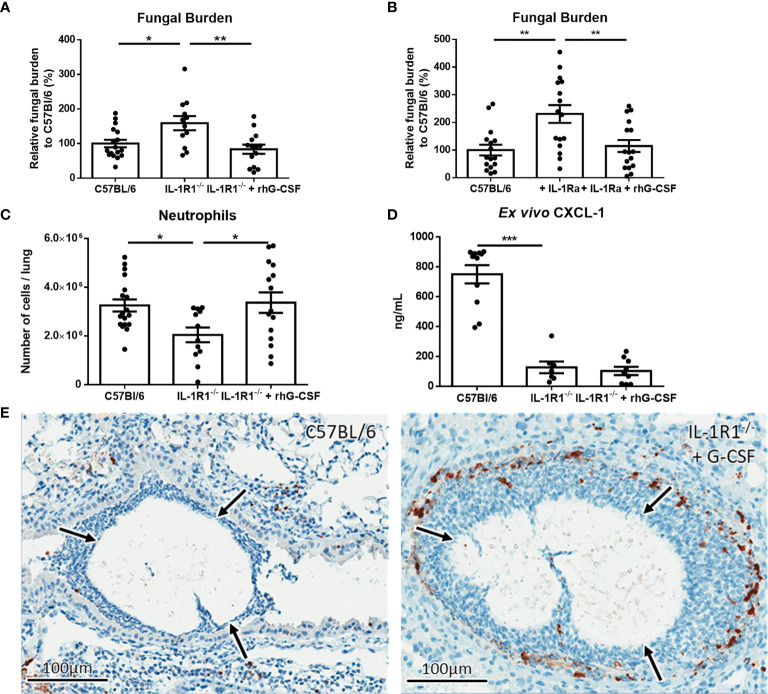
IL-1 receptor-dependent G-CSF secretion increases resistance to *A. fumigatus* airway infection. **(A)** Pulmonary fungal burden of C57BL/6, IL-1R1^-/-^ and IL-1R1^-/-^ mice treated with G-CSF (250µg of rhG-CSF, daily commencing the day of infection) following infection with *A. fumigatus*-containing beads. Fungal burden was measured by galactomannan EIA 3 days after infection. Results represent the mean ± SEM of groups of > 15 mice across 2 experiments, *p ≤ 0.05, **p ≤ 0.01 (one-way ANOVA test). **(B)** Pulmonary fungal burden of C57BL/6 mice treated with IL-1Ra with or without G-CSF (rhG-CSF) as measured by galactomannan EIA 3 days after infection with *A. fumigatus* beads. IL-1Ra was given at a dose of 200 µg/day intraperitoneally commencing 24 hours prior to infection and rhG-CSF was administered as in **(A)**. Results represent the mean ± SEM of groups of > 15 mice across 2 experiments, **p ≤ 0.01 (one-way ANOVA test). **(C)** Total pulmonary neutrophils as determined by flow cytometry analysis of collagenase-digested lungs from the indicated mouse strains 3 days after infection with *A. fumigatus*-containing beads. Results represent the mean ± SEM of groups of n = 16 mice across 2 experiments, **p ≤ 0.01 (one-way ANOVA test). **(D)** Pulmonary *ex vivo* CXCL1 cytokine secretion as determined by ELISA three days after infection. Results represent mean ± SEM of groups of n ≥ 7 mice across 2 experiments, ***p ≤ 0.001 (one-way ANOVA test). Note that the data in this graph for C57BL/6 and untreated IL-1R1^-/-^ mice is reproduced from Figure 4a for ease of comparison as these mice were studied in the same experiments. **(E)** Active caspase 3 immunohistochemistry staining of lungs from C57BL/6 mice infected with *A. fumigatus*-containing beads and IL-1R1-/- mice infected with *A. fumigatus*-containing beads and treated with G-CSF. Note that this staining was done at the same time as those presented in Figure 6 and therefore for continuity it shares the same C57BL/6 image.

To determine if exogenous G-CSF could also enhance the resistance of IL-1R1^-/-^ mice to *A. fumigatus* airway infection, 250μg/kg of G-CSF was administered intraperitoneally to IL-1R1^-/-^ mice and pulmonary fungal burden was measured 3 days after airway infection. Treatment of IL-1R1^-/-^ mice with G-CSF resulted in a reduction in pulmonary fungal burden to levels that were comparable to wild-type mice (**Figure 9A**). G-CSF supplementation of IL-1Ra-treated wild-type mice also enhanced resistance to *Aspergillus* airway infection (**Figure 9B**). G-CSF treatment of IL-1R1^-/-^ mice resulted in an increase in the number of viable pulmonary neutrophils to levels comparable with wild-type mice (**Figure 9C**). This increase in viable pulmonary neutrophils was likely not due to an increase in recruitment as production of the neutrophil chemokine CXCL1 remained low in the IL-1R1^-/-^ mice even after G-CSF treatment (**Figure 9D**). Furthermore, IHC caspase 3 staining of G-CSF treated IL-1R1^-/-^ mice displayed a reduced the degree of caspase 3 activity comparable to wild-type mice mentioned above (**Figure 9E**).

## Discussion

The role of IL-1 cytokines in invasive aspergillosis infections has been the subject of several studies (12, 13, 30, 31). In an immunocompetent high dose mouse model of invasive aspergillosis, IL-1R1^-/-^ C57BL/6 mice exhibited reduced neutrophil recruitment and impaired survival following fungal challenge as compared to wild-type mice (12). Reduced neutrophil recruitment in this model was associated with a significant reduction in the levels of the neutrophil chemokine CXCL1 as well as G-CSF in the BAL of IL-1R1^-/-^ mice (12). Chemokine supplementation increased neutrophil numbers within the lung and partially rescued control of fungal growth. However, the effect of CXCL1 treatment on G-CSF levels was not determined. In a cyclophosphamide and cortisone acetate-treated immunosuppressed mouse model, caspase 1^-/-^ C57BL/6 mice that were deficient in the production of IL-1β were found to have reduced survival (13) and lower levels of pulmonary neutrophils as observed by IHC staining for the neutrophil specific enzyme MPO (13). Although the production of IL-1α in this mouse model was not assessed, it is likely that production of this cytokine was also impaired as previous studies have demonstrated that corticosteroid treatment of alveolar macrophages impairs IL-1α secretion in response to *A. fumigatus* conidia (32). In both of these studies, the reduced numbers of neutrophils within lung tissue of IL-1 pathway deficient mice were proposed to result from a defect in recruitment, however neither study examined neutrophil survival or apoptosis *in vitro* or *in vivo*. Thus, although it is likely that loss of IL-1-dependent neutrophil recruitment plays a role in susceptibility to *A. fumigatus* infection, it is also possible that the reduced neutrophil numbers observed in the lungs of these animals may also reflect reduced neutrophil viability. This hypothesis is consistent with the observations that in the high-dose conidia model, IL-1R1^-/-^ mice produced lower levels of G-CSF during infection and that CXCL1 supplementation only partially restored control of fungal growth (12). In contrast, in the current study, G-CSF treatment had no effect on CXCL1 levels, yet completely restored the ability of IL-1R1^-/-^ mice to control fungal growth *in vivo.* Taken together, our data suggest that the effects of IL-1 receptor signaling on maintaining neutrophil viability plays an important role in mediating host defense during *A. fumigatus* airway infection through maintaining neutrophil viability within the lungs, while IL-1 receptor-dependent CXCL1-mediated neutrophil recruitment is dispensable in this condition. Although these data clearly implicate IL-1R-driven G-CSF in maintaining neutrophil viability, this does not rule out a direct role for IL-1 cytokines or other IL-1 dependent factors in contributing to this process.

The animal model of chronic airway infection differs significantly from those used in prior studies of IL-1α and IL-1β during *A. fumigatus* infection in which mice were infected with either a high dose of conidia, or were immunosuppressed to render them susceptible to infection (12, 13, 21). The model of airway infection used in this study uses immunocompetent mice, and by encapsulating conidia in agar beads, the immune response is generated in response to emerging hyphae or soluble factors elaborated by hyphae (21). Hyphae, but not conidia, of *A. fumigatus* produce the mycotoxin gliotoxin and the secreted polysaccharide galactosaminogalactan, which have both been reported to induce neutrophil apoptosis (33, 34). Thus, it is possible that IL-1R signaling plays a more important role in protecting neutrophils from undergoing apoptosis and death in the presence of significant numbers of hyphae and their secreted products.

Previously, IL-1α, and IL-1β have been reported to induce the production of G-CSF by pulmonary endothelial cells (29, 35, 36). G-CSF promotes bone marrow granulopoiesis, as well as the survival, recruitment, and killing capacity of neutrophils at the site of infection (29, 37–40). Our study expands on the currently known role of G-CSF by demonstrating that G-CSF production is dependent on IL-1 receptor signaling. The mechanism by which G-CSF mediates the inhibition of neutrophil apoptosis requires further study. Although neutrophils from G-CSF knockout mice are not known to undergo accelerated apoptosis, it has been reported that G-CSF can inhibit apoptosis by increasing transcription of anti-apoptotic proteins (41). Our findings that G-CSF treated neutrophils display increased expression of BCL-xL and MCL-1 is in agreement with these reports, and suggests that the upregulation of these anti-apoptotic genes may contribute to the increased viability of neutrophils during infection.

The findings of this study provide insight into the host immune response to non-invasive *A. fumigatus* airway infection. This work establishes a role for the IL-1 pathway in this condition and identifies a novel role for IL-1R-dependent G-CSF production in antifungal defense through inhibiting neutrophil apoptosis and maintaining neutrophil viability.

## Data Availability Statement

The original contributions presented in the study are included in the article/**Supplementary Material**. Further inquiries can be directed to the corresponding author.

## Ethics Statement

The animal studies were reviewed and approved by Animal Care Committees of the McGill University Health Centre (AUP 7609) and the Dartmouth College Institutional Animal Care and Use Committee (Protocol #obar.jj.1).

## Author Contributions

BR designed the study, conducted, analyzed experiments, and wrote the manuscript. ML assisted in performing most animal experiments and most FACS experiments. HO assisted with neutrophil antifungal activity assays and some other experiments. BS assisted with bone marrow neutrophil extractions and ROS experiment set up. AC-C assisted with some animal experiments. RF assisted with histopathological analysis. MS provided valuable direction for the project. JO was supervisor of AC and provided valuable direction for the project. SQ provided valuable direction for the project. DS was supervisor for BR, ML, HO, and BS, conceived the project, co-designed the study, and wrote the manuscript. All authors contributed to the article and approved the submitted version.

## Funding

Funding for the research described in this paper was supported by Canadian Institutes of Health Research (CIHR, https://cihr-irsc.gc.ca/) grants 81361, 123306, and FDN159902, BR has been supported by graduate scholarships from the Fonds de Recherche Quebec Santé (FRQS, http://www.frqs.gouv.qc.ca/). DS has been supported by a Chercheur-Boursier Award from the FRQS. The funders had no role in study design, data collection and analysis, decision to publish, or preparation of the manuscript.

## Conflict of Interest

The authors declare that the research was conducted in the absence of any commercial or financial relationships that could be construed as a potential conflict of interest.
