# Supplementary figures and images for: The IL-1 Receptor Is Required to Maintain Neutrophil Viability and Function During Aspergillus fumigatus Airway Infection
Source: Front Immunol. 2021 Jul 12;12:675294. doi: 10.3389/fimmu.2021.675294 (PMC8312098; doi:10.3389/fimmu.2021.675294)

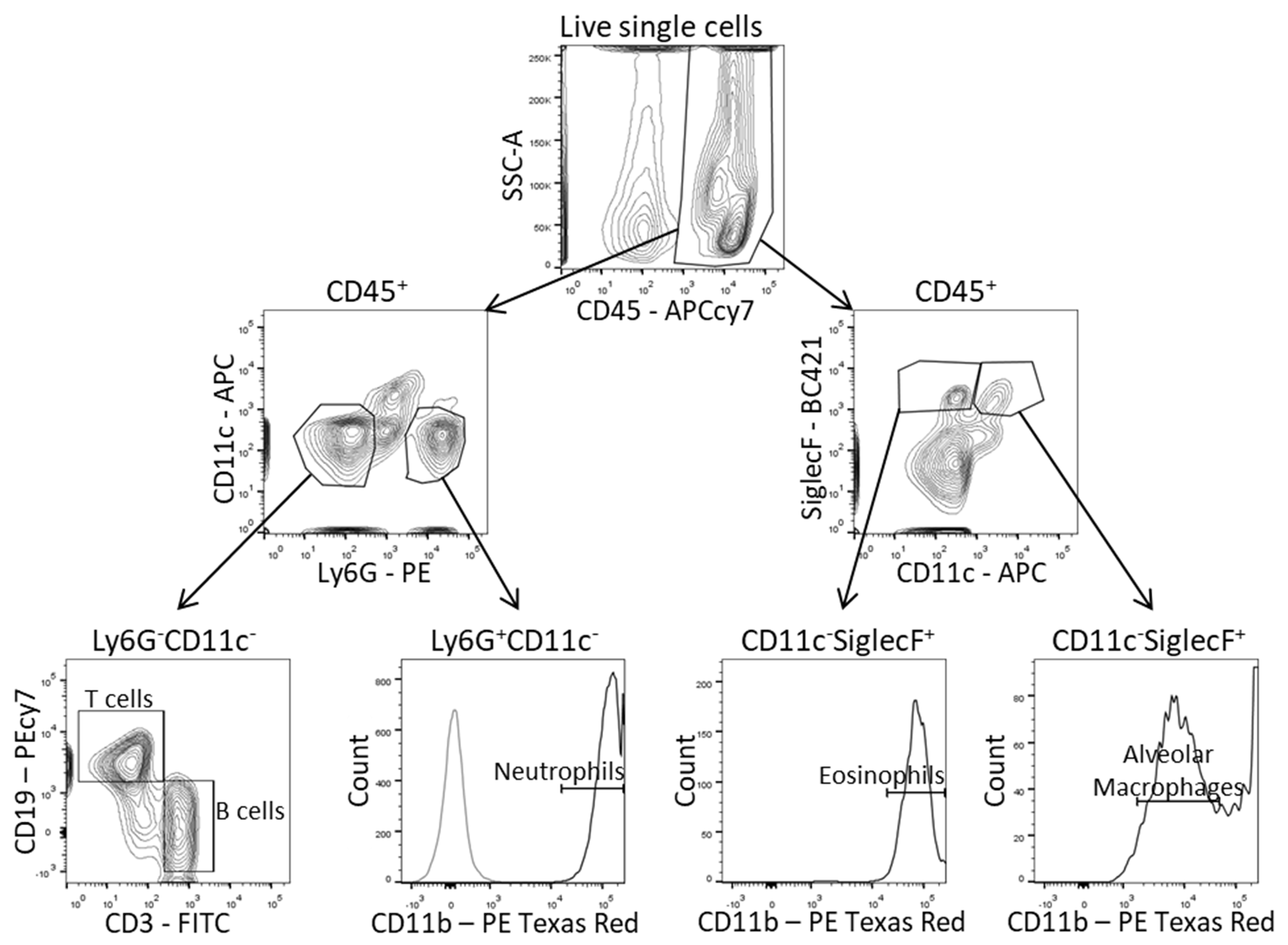

Supplement: Supplementary file 1 [file Image_1.tif]

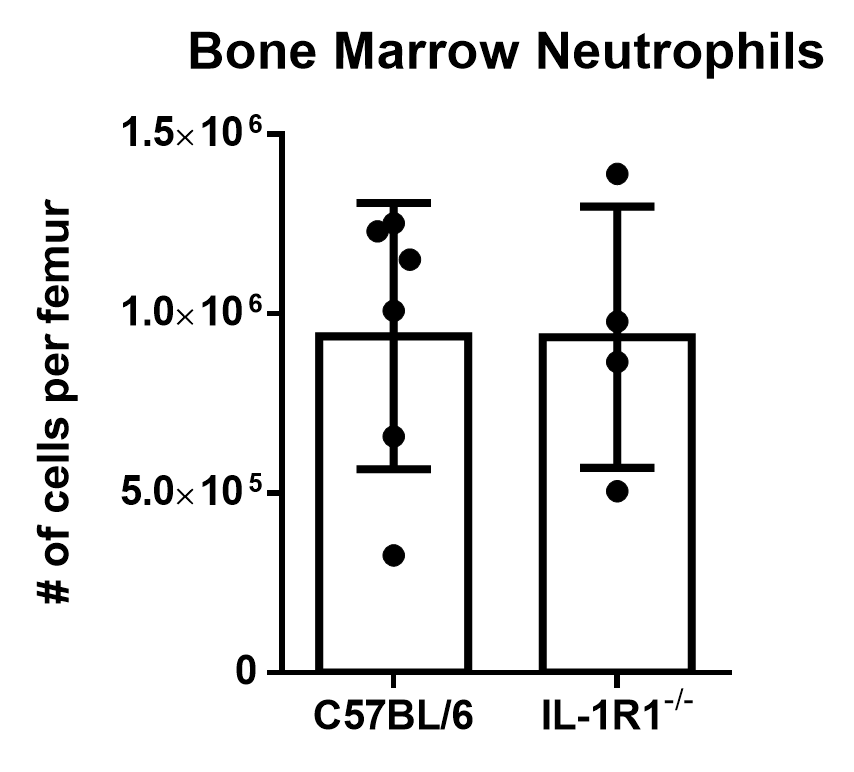

Supplement: Supplementary file 2 [file Image_2.tif]
